# A broadly applicable method to characterize large DNA viruses and adenoviruses based on the DNA polymerase gene

**DOI:** 10.1186/1743-422X-3-28

**Published:** 2006-04-11

**Authors:** Larry A Hanson, Mary R Rudis, Marcia Vasquez-Lee, Roy D Montgomery

**Affiliations:** 1 Department of Basic Sciences, College of Veterinary Medicine, Mississippi State University, P.O. Box 6100, Mississippi State, Mississippi 39762, USA; 2 Department of Pathobiology and Population Medicine, College of Veterinary Medicine, Mississippi State University, P.O. Box 6100, Mississippi State, Mississippi 39762, USA

## Abstract

**Background:**

Many viral pathogens are poorly characterized, are difficult to culture or reagents are lacking for confirmatory diagnoses. We have developed and tested a robust assay for detecting and characterizing large DNA viruses and adenoviruses. The assay is based on the use of degenerate PCR to target a gene common to these viruses, the DNA polymerase, and sequencing the products.

**Results:**

We evaluated our method by applying it to fowl adenovirus isolates, catfish herpesvirus isolates, and largemouth bass ranavirus (iridovirus) from cell culture and lymphocystis disease virus (iridovirus) and avian poxvirus from tissue. All viruses with the exception of avian poxvirus produced the expected product. After optimization of extraction procedures, and after designing and applying an additional primer we were able to produce polymerase gene product from the avian poxvirus genome. The sequence data that we obtained demonstrated the simplicity and potential of the method for routine use in characterizing large DNA viruses. The adenovirus samples were demonstrated to represent 2 types of fowl adenovirus, fowl adenovirus 1 and an uncharacterized avian adenovirus most similar to fowl adenovirus 9. The herpesvirus isolate from blue catfish was shown to be similar to channel catfish virus (Ictalurid herpesvirus 1). The case isolate of largemouth bass ranavirus was shown to exactly match the type specimen and both were similar to tiger frog virus and frog virus 3. The lymphocystis disease virus isolate from largemouth bass was shown to be related but distinct from the two previously characterized lymphocystis disease virus isolates suggesting that it may represent a distinct lymphocystis disease virus species.

**Conclusion:**

The method developed is rapid and broadly applicable to cell culture isolates and infected tissues. Targeting a specific gene for in the large DNA viruses and adenoviruses provide a common reference for grouping the newly identified viruses according to relatedness to sequences of reference viruses and the submission of the sequence data to GenBank will build the database to make the BLAST analysis a valuable resource readily accessible by most diagnostic laboratories. We demonstrated the utility of this assay on viruses that infect fish and birds. These hosts are phylogenetically distant from mammals yet, sequence data suggests that the assay would work equally as well on mammalian counterparts of these groups of viruses. Furthermore, we demonstrated that obtaining genetic information on routine diagnostic samples has great potential for revealing new virus strains and suggesting the presence of new species.

## Background

Many viral pathogens of animals are poorly characterized. To date, if a suspected new virus was identified and the virus could be cultured, morphology, physical characteristics, growth characteristics and antigenic nature were determined. This method of characterization is very time consuming and is limited to culturable viruses (in established cell lines or readily available primary cells). Usually, because of the time and expense, this characterization is limited to viruses that are associated with an important disease. However, a large portion of viruses are either unculturable, difficult to culture or are not associated with a disease of importance to justify in-depth characterization or development of reliable serological reagents. Even with culturable viruses, confirmative diagnosis is often not done because of a lack of diagnostic antibodies or PCR assays. Therefore the development of broad spectrum diagnostic methods that obviate culture are needed as well as methods to bypass the cumbersome traditional methods of characterizing culturable viruses. We addressed this need by using identified sequence conservation between an important group of viral pathogens, the large DNA viruses and adenoviruses. Alignment of the amino acid sequences the DNA polymerase of representatives of Adenoviridae, Poxviridae, Herpesviridae, Iridoviridae, and Baculoviridae reveal two regions that display a high level of conservation [1]. The upstream region showed two different contiguous sequences of conservation with potential for degenerate probe development with the adenoviruses grouping in one, and the herpesviruses, poxviruses, iridoviruses and baculoviruses grouping in the second. One downstream conserved region was shared by all of the virus groups. We designed degenerate primer sets using different upper primers corresponding to the two upstream amino acid sequences and a common lower degenerate primer corresponding to the downstream amino acid sequence. We then validated the assay by using these primers to amplify the fragment of the DNA polymerase gene from case isolates or infected tissues of Ictalurid herpesvirus 1(channel catfish virus-CCV), two iridoviruses-lymphocystis disease virus (LDV) and largemouth bass ranavirus (LBV), avian adenoviruses and avian poxviruses. We were able to readily obtain the expected products from all of these viruses except the avian poxviruses. In order to obtain the sequence for the avian poxvirus, DNA extraction methods were optimized and new primers were developed. The poxvirus sequence was finally obtained and the difficulty was likely due to secondary structure of the PCR product and/or competition by aberrant products.

## Results

### Sequence alignment

**Figure 1:**
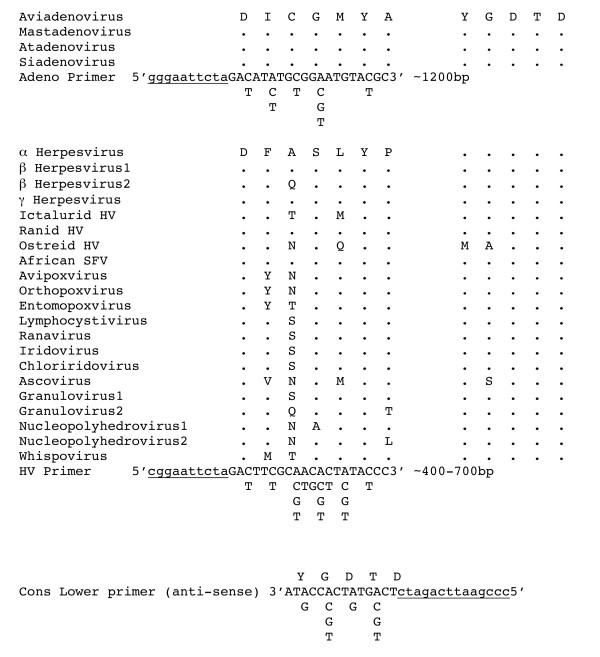
**Amino acid alignment of conserved regions of the DNA polymerase of selected viruses and representative primers designed for this study**. Upper primers are displayed 5'-3' and the lower primer is displayed 3'-5'. Underlined lower case nt represent 5' regions with no homology to coding region. The ~ 1200 bp and 400–700 bp following the adeno and HV primers indicate the respective distance to the region with homology to the lower primer. Represented sequences are: Aviadenovirus – fowl adenovirus A [GenBank:NP_043878], Mastadenovirus-human adenovirus C [GenBank:NP_040516], Atadenovirus-duck adenovirus 1 [GenBank:NP_044702], Siadenovirus-frog adenovirus [GenBank:NP_062435], α Herpesvirus-human herpesvirus 1 [GenBank:NP_044632], β Herpesvirus1-human herpesvirus 5 [GenBank:P08546], β Herpesvirus2-human herpesvirus 6 [GenBank:NP 042931], γ Herpesvirus-human herpesvirus 4 [GenBank:NP_039908], Ictalurid HV-Ictalurid herpesvirus 1 [GenBank:NP_041148], Ranid HV-ranid herpesvirus 1 [GenBank:AAD12269], Ostreid HV-Ostreid herpesvirus 1 [GenBank:AAS00986], African SFV-African swine fever virus [GenBank:NP_042783], Avipoxvirus-fowlpox virus [GenBank:NP_039057], Orthopoxvirus-Vaccinia [GenBank:NP_063712], Entomopoxvirus-Melanoplus sanguinipes entomopoxvirus [GenBank:NP_048107], Lymphocystivirus-lymphocystis disease virus 1 [GenBank:NP_078724], Ranavirus-frog virus 3 [GenBank:YP_031639], Iridovirus-Invertebrate iridescent virus 6 [GenBank:NP_149500], Chloriridovirus-Invertebrate iridescent virus 3 [GenBank:CAC84133], Ascovirus-Heliotis virescens ascovirus [GenBank:AJ312696]. Granulovirus1-Cryptophlebia leucotreta granulovirus [GenBank:NP_891948], Granulovirus2-Xestia c-nigrum granulovirus [GenBank:AAF05246], Nucleopolyhedrovirus1-Lymantria dispar nucleopolyhedrovirus [GenBank:NP_047720], Nucleopolyhedrovirus2-Orgyia pseudotsugata multicapsid nuclear polyhedrosis virus [GenBank:Q83948], Whispovirus-shrimp white spot syndrome virus [GenBank:AAK77696].

The deduced amino acid sequences of DNA polymerases encoded by representative members of several subgroups of DNA viruses of animals (herpesviridae, poxviridae, adenoviridae and baculoviridae) had been aligned [1] and two areas of conservation were identified. We analyzed these regions in additional viruses including Ostreid herpesvirus 1, African swine fever, iridoviridae, an ascovirus, and whitespot disease virus of shrimp. We were looking for two highly conserved regions of consecutive amino acids (aa), spaced 70 – 400 aa apart. This would allow the design of degenerate PCR primers that would cover a large number of viruses and yield a useful, easily amplified product (large enough for sequence comparisons yet small enough for efficient PCR). There was considerable variation in the deduced amino acid sequences between families. Several small regions of conservation were identified. Only one region with conservation of at least 5 consecutive amino acids was found among nearly all sequences evaluated. This was the YGDTD sequence previously described [1]. The only differences among viruses analyzed were a serine instead of the glycine at the second amino acid of the Ascovirus and methionine, alanine instead of tyrosine, glycine as the first two amino acids in Ostreid herpesvirus 1. Approximately 400 to 700 bp upstream of this region was a portion that was relatively conserved in all of the virus groups except Adenoviridae but a region approximately 1200 bp upstream within adenoviridae was conserved (Figure 1). Therefore we designed one degenerate downstream primer to be used for all large DNA viruses of vertebrates (Cons lower primer-5'cccgaattcagatcTCNGTRTCNCCRTA3' N = A/C/G/T, R = A/G) and two degenerate upstream primers, one representing Adenovirus (Adeno primer-5'gggaattctaGAYATHTGYGGNATGTAYGC3' Y = T/C, H = A/C/T) and the other based on herpesvirus sequences but representing the other large DNA viruses of vertebrates (HV primer-5'cggaattctaGAYTTYGCNWSNYTNTAYCC3' S = C/G, W = A/T) (Figure 1). We added additional sequence to the 5' ends to improve amplification properties with lower primer having 14 nucleotides (nt) of additional sequence and the upper primers having 10 nt of additional sequences (indicated above by lowercase letters). The additional sequences also provided *Eco*RI and *Bgl*II restriction enzyme recognition sites to the lower primer and *Eco*RI and *Xba*I recognition sites to the upper primers that could be used for cloning purposes. When the amino acid sequences used to design the Adeno primer and the cons lower primer were used together in BLAST analysis against the GenBank non-redundant database, only adenovirus DNA polymerase genes were found with substantial identity. When the amino acid sequences used to design the HV primer and cons lower primer were used together in BLAST analysis against the GenBank non-redundant database, a wide variety of DNA polymerase genes were found to contain identical or nearly identical sequences. These included the DNA polymerase genes of the virus groups listed in figure 1 as well as a large number of members of the Phycodnaviridae, Archea, plants, fungi, ciliates, plasmodia, nematodes, echinoderms, insects, and vertebrates. The vertebrate host tissues and cells represented the strongest potential source of unwanted PCR products but the vertebrate DNA polymerase genes contain introns making the DNA polymerase products from the host genomic DNA much larger than from viral genomic DNA. The predicted PCR product from the mouse genome would be 2622 nt [GenBank:NC_000073] and for 3556 nt for *Danio rerio *[GenBank:NC_007114].

### Application of primer sets to representative viruses

We tested the designed primer sets on avian and fish case isolates representing the most common DNA viruses of vertebrates that contain DNA polymerase genes: herpesviridae, iridoviridae, poxviridae and adenoviridae.

**Figure 2:**
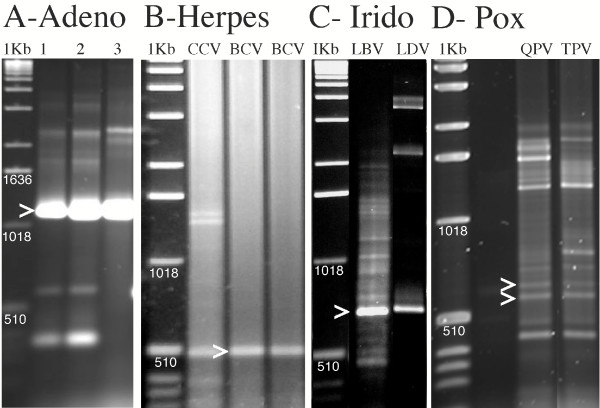
**Agarose electrophoretic profiles of amplification products from DNA polymerase targeted-degenerate PCR from avian adenovirus samples (A], catfish herpesvirus samples (B), fish iridovirus samples (C) and Avian Poxvirus samples (D)**. The > indicates bands that were evaluated by sequencing. A-chicken adenovirus isolates CELO-lane 1, case 162-lane 2, case 1422b-lane 3 using adenovirus upper and consensus lower primers. The products of interest were 1200 bp. B-lanes designated CCV and BCV represented the type specimen (Auburn clone A) and the blue catfish isolates respectively produced 465 bp bands using HV and cons lower primers. C-Largemouth bass ranavirus type specimen-LBV, and lymphocystis disease virus case (infected fin tissue) LDV produced 695 bp and 662 bp bands, respectively, using HV and cons lower primers. D Avian Poxvirus from Quail (QPV) and turkey (TPV) produce multiple bands using the HV and Cons lower primers on DNA extracts from infected chicken chorioallantoic membranes.

The DNA polymerase PCR was performed on three adenovirus isolates from infected chicken primary fibrobasts using the Adeno and Cons lower primers. All three gave strong single bands at the expected 1200 bp size (Figure 2A). Direct sequencing done on the excised products using upper and lower primers respectively demonstrated that the products of our chicken embryo lethal orphan virus (CELO, fowl adenovirus 1) strain of Fowl adenovirus A, and case 162 were similar and case 1422 demonstrated some divergence from the other two. The products were cloned and sequenced using vector primers and internal sequencing primers to resolve the entire fragments. The sequences of the first two adenovirus samples were identical to the published sequences of the represented region of CELO [GenBank:U46933]. The case 1422 isolate sequence [GenBank:DQ159938] was most similar to the respective region of fowl adenovirus 9 strain of Fowl adenovirus D [GenBank:AF083975] of adenovirus DNA polymerases that had been sequenced. The 1145 nt region between the primers showed 68.5% identity at the nucleotide level and a corresponding 66.4% identity at the amino acid level.

As a test for utility of DNA polymerase PCR on herpesvirus samples, DNA was amplified from virus isolates from two cases of diseased from blue catfish (*Ictalurus furcatus*) using the HV primer with the Cons lower primer. These isolates were designated as "blue catfish virus" (BCV) because they were shown to be herpesviruses by electron microscopy and produced similar cytopathic effect on CCO cells as CCV but would replicate in the Chinook salmon embryo cell line (CHSE 214) where as CCV would not. The DNA for these samples and the type isolate of CCV were isolated from infected CCO cells. All three produced a distinct 465 bp band after DNA polymerase PCR (Figure 2B lanes CCV and BCV represent type virus and blue catfish isolate respectively). Cloning and sequencing this fragment from the two blue catfish isolates [GenBank:DQ159941] demonstrated 100% nt identity between each other and 97.7% nt identity to CCV (10 nt difference in 439 nt) with 100% amino acid identity. This suggests that the blue catfish isolate is a strain of CCV and our data provides strong evidence for a broader host range for CCV.

To test the utility of the primer set for Ranavirus genus of the Iridoviridae, we used the HV-Cons lower primer set on DNA from two isolates of largemouth bass ranavirus (LBV). One was the type virus, the other was a case isolate from a diseased largemouth bass in Mississippi. Both were cultured on fathead minnow cells and both yielded 695 bp products (figure 2C-LBV). The PCR products of both isolates were identical [GenBank:DQ159940]. No previous LBV DNA polymerase sequence had been submitted to GenBank. The highest BLAST scores were to the DNA polymerase genes of tiger frog virus [GenBank:AAL77804.1] and frog virus 3 [GenBank:AAT09720.1]. Simple alignment of the 641 nt between the primers demonstrated 76.6% (491 nt) and 76.3% (490 nt) identity to tiger frog virus and frog virus 3 respectively. The deduced 213 aa sequence demonstrated 80.28% (171 aa) identity to each.

To test the utility of this assay for Lymphocystis disease virus (LDV) genus of Iridoviridae and the use of this assay directly on tissues, DNA was extracted from pathognomonic lymphocystis disease lesion on the caudal fin of a largemouth bass. The 662 bp product was amplified using the HV primer with the Cons lower primer (figure 2C, Lane LDV). Sequence analysis [GenBank:DQ159939] demonstrated that the highest similarity of the 608 nt region between the primers was the corresponding region from a LDV isolated from flounder in China [GenBank:AY380826.1] with 75.16% (457 nt) identity. The deduced 202 aa sequence demonstrated 77.7% (157 aa) identity. In comparison the same region from LDV 1 from flounder in North America [GenBank:L63545.1] demonstrated 70.68% nt identity and 69% deduced aa identity. Our data suggests that the largemouth bass isolate of LDV may be a different species from the two previously characterized LDV isolates.

**Figure 3:**
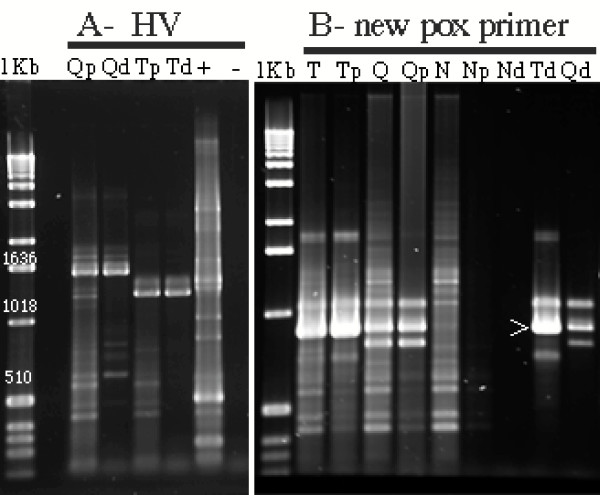
**Agarose electrophoretic profiles of amplification products from DNA polymerase targeted-degenerate PCR on quail and turkey isolates of avian poxvirus using the consensus lower primer with HV upper primer (A), and the poxvirus specific primer (B)**. Lanes are designated Q, T and N for quail virus, turkey virus and no virus infected chicken chorioallantoic membrane, respectively, + indicates the positive control (CCV DNA) and – indicates a negative water control, 1 Kb = 1 Kb ladder (Invitrogen), lower case letters indicate extraction protocols with no designation being a total DNA extraction from the tissue, p indicating pelleted sample (the virus was filtered through a 0.45 μm filter and pelleted at 20,000 × g before DNA extraction) and d indicating DNase treament (the pelleted sample was resuspended and DNase treated before DNA extraction). The > indicates product that was sequenced.

To test the utility of the assay on Poxviridae we obtained avian poxvirus isolates from quail and turkey. DNA samples extracted from infected chicken chorioallantoic membrane tissue were used to performed the degenerate PCR assays. We generated many different bands in these assays (Figure 2D, lanes QPV and TPV for quail and turkey isolates respectively) so two bands closest to the expected 600 bp these were re-amplified, cloned and sequenced. BLAST analysis demonstrated that both of the sequences were derived from chicken genomic DNA. To reduce host genomic DNA contamination we filtered the tissue homogenate through 0.45 μm filters and DNAse treated the samples before nucleic acid extraction. These treatments greatly simplified the banding pattern (compare Figure 2D with Figure 3A, lanes Qp and Tp were filtered and Lanes Qd and Td were DNAse treated). Cloning and sequencing of 3 bands all revealed fowl pox sequences but none were the DNA polymerase gene. We theorized that the problem may have been due to excessive mis-matches. The *Chordopoxvirinae *upstream amino acid sequence was DYNSLYP verses DFASLYP this would result in 3 nt mis-matches at the 5' end of the upstream target. When we calculated the degeneracy required to cover *Chordopoxvirnae*, and Herpesviridae, we would have a degeneracy of 32768, which was excessive. However, if we made 8 separate degenerate primers and combined them we could eliminate nucleotide combinations at the serine and leucine encoding sites that do not encode the desired amino acid. This resulted in a primer mixture with a degeneracy of 3456, lower than the HV primer. We tried the new primer set. Cloning and sequencing of three bands close to the expected 600 bp size revealed avipoxvirus sequences but no DNA polymerase gene. We re-evaluated potential primers for poxviruses. Alignment using *Chordopoxvirinae *DNA polymerase aa sequences identified an alternative upstream primer. The best contiguous sequence near the previous upstream primer target was YCIHDAC. PCR using the respective degenerate upstream primer 5'TAYTGYATHCAYGAYGCNTG'3 and the cons lower primer generated the expected 882 bp band from both quail and turkey isolates (figure 3B). This primer set generated the expected band on nucleic acid extracts from the tissue, tissue homogenate that had been filtered and virus pelleted and pelleted virus that was treated with DNAse before extraction but not from control tissue. Figure 3 clearly demonstrates the advantage of concentrating the virus and treating the sample with DNAse to eliminate non-specific bands [compare lanes T, Q and N (tissue extract) to Tp, Qp and Np (pelleted virus extract) and Td Qd and Nd (pelleted and nuclease treated before extraction)]. The DNA polymerase gene fragment product from the turkey isolate was confirmed by sequencing. It exactly matched that of fowlpox virus [GenBank:NC_002188].

Aberrant products that were sequenced during this research generally provided only small regions of similarity to known DNA sequences, often having some similarity to microsatellite sequences or to putative retrovirus provirus sequences of various genomes. The most common of the aberrant products generated from the poxvirus research was a portion of ORF FPV115 an Ankyrin repeat gene family protein [2].

## Discussion

The use of degenerate primers to DNA polymerase gene to amplify a DNA fragment and identify the presence of DNA viruses have been used by many researchers for specific research projects or to characterize a virus associated with a specific disease. Also, the amino acid sequences of DNA polymerase of many large DNA viruses have been compared to evaluate virus relationships and compared to the DNA polymerases of other organisms to hypothesize the relationship and origin of this family of molecules[1,3,4]. The regions targeted with our degenerate primers were identified by the original alignment of Ito and Braitwaite [1] and overlap regions designated as regions 2 and 4 by Villarreal et al [4]. VanDevanter et al [5] used degenerate primers to the DNA polymerase gene of mammalian and avian herpesviruses to identify unknown herpesviruses and were successful in identifying gene sequences of herpesviruses from several species of mammals. They were however, unsuccessful in amplifying the DNA fragment from CCV and did not evaluate the utility of there primers on other families of DNA viruses. Our primers targeted similar regions as two of their primers. Our HV primer targeted was similar to their DFA primer targeting the DFASLYP sequence. The 3' (gene specific) portion of our sequence being 5'GAYTTYGCNWSNYTNTAYCC3' compared to 5'GAYTTYGCNAGYYTNTAYCC3'. Their IYG downstream primer targeted IYGDTDSV with the corresponding degenerate primer being 5'CACAGAGTCCGTRTCNCCRTADAT3'. Our Cons lower primer targeted YGDTD with the gene specific portion of the degenerate primer being TCNGTRTCNCCRTA3'. Ehlers et al [6] utilized similar primers as VanDevanter et al [5] but they included deoxyinosines at sites with a degeneracy of 3 or more. The differences in the primers allows all codons for serine to be represented in the HV primer and the narrower target of our cons lower primer accounts for different amino acids flanking the YGDTD sequence in non-herpesvirus targets. Our objective in this study was to develop a broad spectrum method that could be used to characterize most large DNA viruses including those in which the virus type is poorly defined, those that have not been cultured and those that come from a host that is phylogenetically distant from the hosts of well characterized members of the DNA viruses. This goal necessitated the use primers with a high degree of degeneracy. Yet, most of the samples readily yielded the desired products even when there were up to two nt mismatches (with CCV). Our success is likely due to the high copy number of viral genomes present on our DNA extracts. The specific product yield was substantially increased when the filtration step, virus concentration and DNase treatment were added to the tissue/cell extraction procedure. We believe that these steps substantially reduced the complexity of the target and improved the efficiency of the degenerate primer PCR.

We chose to use generic primer sets with more degeneracy rather than family specific primer sets because they would be more readily used in a diagnostic environment. The use of limited generic primer sets allows for the application of the assay before the disease agent is as extensively characterized. The use of generic primers has the added advantage of covering most known variants, this minimizes the effect of unique species-specific sequences within a "conserved" region that often occur in virus families. We demonstrated the utility of our assay on defined virus isolates and virus samples that had not been characterized of four families of DNA viruses. Furthermore, we demonstrated that the methodology was directly applicable to infected tissues. The PCR products generated from this assay are sufficiently long for detailed sequence comparison and for the development of specific PCR primers for diagnostic and research applications.

Our difficulty in generating a fragment from the poxvirus sample was unexpected because the degenerate primers were matched to that sequence. The alternate primer set worked very well and because the secondary structure may cause similar problem with poxviruses the use of the pox specific primer set may be warranted when a poxvirus is suspected. In the process of optimizing the procedure for the poxviruses, we found that the use of DNAse treatment of the tissue/cell homogenate before DNA extraction greatly improved the specificity of the assay. Even with the poxvirus, the primer sets that did not amplify the DNA polymerase did amplify poxvirus sequences and sequence analysis of non-targeted sequences may be of use in characterizing a newly discovered virus. The use of nuclease treatment in conjunction with sequence independent single primer amplification has been very successful for identifying unknown viruses in serum samples [7]. The advantage of using degenerate PCR is that the product obtained is a fragment of a specific gene, which simplifies comparisons to known orthologs for phylogenetic placement of the virus.

The assay that we developed is being quickly adapted in fish virology community to evaluate suspected cases of virus infected tissues or to characterized culturable viruses. The primers were successfully used to amplify the DNA polymerase gene products from 3 cyprinid herpesviruses, [8,9](personal communication Janet Warg, Diagnostic Virology Laboratory, National Veterinary Services Laboratories, Ames, Iowa). This assay has been used to characterize 7 herpesviruses from sturgeon [10].

## Conclusion

In this report, we use a defined region of a gene common to all large DNA viruses to develop a general diagnostic method that is broadly applicable to a wide spectrum of viruses. We demonstrated the utility of this system on cell culture isolates and on infected tissues of four major groups of DNA viruses; the Poxviridae, Herpesviridae, Adenoviridae and Iridoviridae. Although the assay was applied to a small sample of the viruses (1–3 examples per group), they represented diverse virus families and included up to 2 amino acid mismatches in the upstream target region. Success by other laboratories and amino acid sequence analysis of DNA polymerases of other members of these groups supports the broad applicability of this assay to the large DNA viruses and adenoviruses of vertebrates. The Phycodnaviridae found in algae, Baculoviridae and Ascoviridae found in arthropods and the herpesviruses of mollusks should also be amenable to this procedure with modified primers. This assay will not work on RNA viruses and DNA virus types that do not have a DNA dependant DNA polymerase gene such as Hepadnaviridae, Circoviridae, Parvoviridae, Papillomaviridae and Polyomaviridae. We demonstrated the benefit of using the defined region for matching case isolates to species that have been previously sequenced and demonstrated a useful scenario for identifying species of which the DNA polymerase gene have not been previously sequenced. As this target fragment of more species of DNA viruses are sequenced, BLAST and GenBank will prove to be a utilitarian software and database for virus diagnostic work that is readily available to all diagnostic and research laboratories. The advantages of the selected target for diagnostic use are: it is sufficiently small that the product can be efficiently generated, yet, there are regions that are highly conserved allowing general placement of unknown viruses into families and there are regions of sufficient variation to allow the development of specific PCR primers. The use of DNAse pretreatment in the extraction protocol simplified the substrate and to allow effective amplification even with highly degenerate primers.

Our optimized protocol is: 1. Disrupt the cells/tissue to release the virions. 2. Pellet the cellular debris by centrifugation at 1000 × g for 5 min. 3. Filter the supernatant through a 0.45 μm filter. 4. Concentrate the virus from the filtrate by centrifugation at 21,000 × g for 30 min. 5. Resuspend the pellet in a small volume of water and DNase treat the suspension to reduce cellular DNA. 5. Extract the DNA. 6. Run degenerate PCR. 7. clone predominant bands of the appropriate sizes (450–800 bp for the HV primer and 1200 bp for the Adenovirus primer). 8. Sequence the products and use BLASTx to compare the translated sequence to deduced amino acid sequences in GenBank. Variations that are helpful are (1) to use larger amounts of cells or tissues in samples that are suspected of having low numbers of virions and (2) to run a negative control of non-infected tissue when multiple weak bands are produced to distinguish cellular products from virus product candidates.

## Methods

### Virus sources

All diagnostic case samples were submitted to the Mississippi State University, College of Veterinary Medicine Diagnostic Laboratory. Virus isolates were obtained from infected diagnostic samples by homogenizing the tissues in serum free medium (SFM) or tryptose phosphate broth (TPB) at the rate of approximately 1 part tissue to 5 parts TPB (vol:vol), passed through a 0.20 μm filter, and diluted 1:10 (vol:vol) in SFM or TPB containing penicillin (100 units/ml) and streptomycin (100 μg/ml).

Avian samples were inoculated onto 24-hour-old monolayers of chicken embryo kidney (CEK) cells or onto the chorioallantoic membrane (CAM) of embryonated eggs. For CAM culture, eleven-day-old embryonated eggs from a commercial specific-pathogen free (SPF) source (Hy-Vac, Inc., Gowrie, IO) were inoculated via the CAM route using 0.2 ml of the antibiotic-treated sample/egg. The eggs were sealed, incubated at 37°C, and candled daily. Those eggs containing live embryos 6 days later, were opened and the CAMs in the area of inoculation were examined. CAMs containing plaques or similarly-suspicious lesions were harvested and pieces placed into McDowell's fixative for histological examination. The rest of the affected membranes were held frozen (-60°C). Histological evaluation identified CAM samples as avian poxvirus-infected when epithelial hyperplasia and eosinophilic intracytoplasmic inclusions were demonstrated [11]. Inoculated CEK cells were observed daily. At 2, 4, and 6 days postinoculation (PI), aliquots of cells and supernatant were harvested and frozen at -70°C. These aliquots were pooled and served as subsequent inocula for two additional 6-day passages. Harvests from any passage showing evidence of "round-cell" cytopathology were tested against adenovirus-specific antiserum (SPAFAS, Inc., Norwich, Conn.) in an agar-gel precipitin (AGP) test. Avian poxvirus isolate 4905 was from a quail in 1984 and isolate M6959 from a wild turkey near Jackson Mississippi in 1989. The avian adenovirus isolates, were chicken embryo lethal orphan (CELO) virus that was used as the positive control for the AGP test, case isolates 162 and 1422b were from diseased chickens in commercial broiler operations in Mississippi in 2002.

Channel catfish virus was either the type strain (Auburn clone A-American Type Culture Collection) or from diagnostic cases S98-675 and S98-697 from diseased blue catfish *Ictalurus furcatus *from a commercial catfish production pond near Inverness, Mississippi in 1998. Virus was propagated by infecting monolayers of channel catfish ovary cell line at approximately 0.1 plaque forming units per cell and freezing the cells and medium when the entire cell sheet was involved in cytopathic effect (CPE). Largemouth bass virus (LMBV) case isolate was from a diseased largemouth bass (*Micropterus salmoides*) found in a private use pond near Brandon, Mississippi (case C01-170) in 2001. The type specimen was from the first described case of LMBV from the Santee-Cooper reservoir, South Carolina [12] and was provided by Dr. V. Greg Chinchar (University of Mississippi Medical Center, Jackson MS). Both were cultured on the Fathead Minnow (FHM) cell line. The lymphocystis disease virus sample was extracted from fin lesions from largemouth bass with lymphocystis disease (case C02-033, found in a commercial catfish production pond near West Point, Mississippi in 2002).

### Sample preparation

Virus from infected cell cultures in 25 cm^2 ^flasks were released from the cells by serial freeze thaw cycles, the debris was centrifuged out at 1000 × g for 5 min then virus was concentrated out of the supernatant by centrifugation at 21,000 × g for 30 min in a microfuge. The pellet was suspended in 80 μl of water. When tissues were evaluated, the DNA was either extracted directly from a 50 mg tissue sample or virus was concentrated from the sample. This was done by homogenizing approximately 200 mg of the tissue sample in 2.25 ml of serum free cell culture medium, centrifuging the sample at 1000 × g for 5 min and concentrating the virus out of the supernatant as described above. The filtration variation to the protocol involved filtering the supernatant of the 1000 × g centrifugation step with a 0.45 μm syringe filter then proceeding to the virus concentration step. The DNase variation on the protocol involved adding 20 μl of 10 × buffer and 100 μl of RQ1 DNAse (Promega) to the 80 μl of concentrated virus and incubating it at 37°C for 2 hours. This was followed by the addition of 20 μl of stop buffer (20 mM EGTA, pH 8.0) and a 10 min incubation at 65°C to inactivate the DNAse. DNA was isolated using Puregene genomic DNA isolation system (Gentra, Minneapolis, MN). The sample was suspended in 600 μl Puregene cell lysis solution containing 60 μg proteinase K, incubated overnight at 50°C then the DNA was isolated according to the manufacture's suggested procedure. DNA was quantitated using UV spectrophotometry (GeneSpec I, Hitachi Software Engineering Company LTD, Japan).

### Degenerate PCR and cloning

PCR consisted of approximately 100 ng of template DNA, 20 pmole of each primer, 4 μl 10 mM dNTP, 5 μl 10 × buffer, 2.5 U Taq polymerase mix (Fisher Scientific) in 50 μl reactions. PCR used the appropriate forward primer and the consensus reverse primer (Figure 1). The reaction conditions were: 93°C, 1 min for one cycle followed by 93°C, 30 sec; 45°C, 2 min; 72°C 3 min for 35 cycles followed by a single cycle at 72°C for 4 min. Product was evaluated by electrophoresis on 1.5% agarose gels followed by staining with GelStar nucleic acid gel stain (BioWhittaker Molecular Applications, Rockland, ME) and UV transillumination (ChemiImager 5500, Alpha Innotech Corporation, San Leandro, CA). Bands of interest were excised from the gel and the DNA was recovered using GenElute Agarose Spin Columns (Supelco, Bellefonte, PA). The product was cloned into plasmid pT7blue using the Perfectly Blunt Cloning Kit (Novagen) or the plasmid pCR4-TOPO using the TOPO TA cloning kit (Invitrogen). Selected candidate clones were evaluated for a DNA insert of the appropriate size using colony PCR (as described in the Perfectly Blunt or TOPO TA cloning kit). Then plasmid was purified for sequencing from 1 ml cultures using the QIAQUIK plasmid purification kit (Qiagen).

### Sequencing

PCR products from all virus samples except the adenovirus isolates were cloned and then sequenced. Sequencing was performed on both strands of at least three clones from each product using vector specific forward and reverse sequencing primers in with the ABI PRISM™ Big Dye Terminator Cycle Sequencing Ready Reaction Kit (Applied Biosystems, Foster City, CA) and by the use of the ABI PRISM™ 310 Genetic Analyzer (Applied Biosystems). A modification of this was the use of direct sequencing of the Adenovirus PCR product using 500 ng of template excised from an agarose gel and 3.2 pmole of upper or lower primer, respectively. Additional sequencing was done on cloned PCR products from the adenovirus samples using vector specific forward and reverse sequencing primers, a lower strand primer-ACGATTTSAGTGCCTTCGTAGATG and a upper strand primer-CATCTACGAAGGCACTSAAATCGT. Data were assembled using MacDNASIS and sequences were edited by manual comparison of overlapping electropherograms (Version 3.7, Hitachi Software Engineering America, Ltd., South San Francisco, CA). The DNA sequence data were analyzed and amino acid sequences deduced using MacDNASIS. Related amino acid sequences were identified using BLASTx [13]. ClustalX [14] was used to align the deduced amino acid sequences of the DNA polymerase fragment.

## Competing interests

The author(s) declare that they have no competing interests.

## Authors' contributions

LAH obtained fish case isolates, performed the DNA sequence comparisons, designed the primers and was the contributing author. MRR performed all fish virus cell culture, DNA extraction, PCR protocol development, and most of the PCR, cloning and sequencing. MV-L cloned and sequenced the adenovirus samples and the type specimen of LMBV. RDM obtained avian diagnostic isolates and produced the virus for these assays. All authors contributed to the writing of this manuscript.
